# Continuous irrigation after pancreatectomy: a systematic review

**DOI:** 10.1007/s00423-023-03070-5

**Published:** 2023-09-02

**Authors:** Ilaria Pergolini, Florian Scheufele, Elke Demir, Stephan Schorn, Helmut Friess, Güralp O. Ceyhan, Ihsan Ekin Demir

**Affiliations:** 1 Department of Surgery, Technical University of Munich, Klinikum rechts der Isar, Ismaninger Straße 22, 81675 Munich, Germany; 2 German Cancer Consortium (DKTK), Partner Site, Munich, Germany; 3 CRC 1321 Modelling and Targeting Pancreatic Cancer, Munich, Germany; 4 Department of General Surgery, HPB-Unit, School of Medicine, Acibadem Mehmet Ali Aydinlar University, Istanbul, Turkey; 5 Else Kröner Clinician Scientist Professor for Translational Pancreatic Surgery, Munich, Germany

**Keywords:** Continuous irrigation, Pancreatic fistula, Pancreatic surgery

## Abstract

**Purpose:**

Prevention and management of postoperative pancreatic fistula (POPF) after pancreatic resections is still an unresolved issue. Continuous irrigation of the peripancreatic area is frequently used to treat necrotizing pancreatitis, but its use after elective pancreatic surgery is not well-known. With this systematic review, we sought to evaluate the current knowledge and expertise regarding the use of continuous irrigation in the surgical area to prevent or treat POPF after elective pancreatic resections.

**Methods:**

A systematic search of the literature was conducted according to the PRISMA 2020 guidelines, screening the databases of Pubmed, Scopus, Web of Science, and Ovid MEDLINE. Because of the heterogeneity of the included articles, a statistical inference could not be performed and the literature was reviewed only descriptively. The study was pre-registered online (OSF Registry).

**Results:**

Nine studies were included. Three studies provided data regarding the prophylactic use of continuous irrigation after distal and limited pancreatectomies. Here, patients after irrigation showed a lower rate of clinically relevant POPF, related complications, lengths of stay, and mortality. Six other papers reported the use of local lavage to treat clinically relevant POPF and subsequent fluid collections, with successful outcomes.

**Conclusion:**

In the current literature, only a few publications are focused on the use of continuous irrigation after pancreatic resection to prevent or manage POPF. The included studies showed promising results, and this technique may be useful in patients at high risk of POPF. Further investigations and randomized trials are needed.

**Supplementary Information:**

The online version contains supplementary material available at 10.1007/s00423-023-03070-5.

## Introduction

The prevention and management of the postoperative pancreatic fistula (POPF) represent still a big challenge in pancreatic surgery. POPF occurs in 3–45% of cases and is the main cause of postoperative morbidity in pancreatic surgery, prolonged hospital stay, and increased health care costs [1]. Great efforts have been made to prevent and mitigate the occurrence and severity of POPF resulting in plenty of publications. Here, several studies have sought to identify risk factors and predictors for POPF to improve preoperative patients’ risk stratification and postoperative management [2, 3]. Many other prospective and retrospective studies, randomized controlled trials, systematic reviews, and meta-analyses have analyzed and compared different surgical techniques, such as different entero-pancreatic anastomosis (i.e., pancreaticogastrostomy vs. pancreaticojejunostomy, with or without pancreatic stent) [4–6] or transection procedures (i.e., stapler vs. hand-suture) [7], with controversial results. Other studies have questioned the use of somatostatin analogues [8, 9] and, the use and management of abdominal drains (i.e., drain vs. no drain, or early vs. late drain removal) [10–12]. Further studies also attempted to test and promote alternative procedures, such as the endoscopic injection of botulin toxin into the sphincter of Oddi [13, 14] or techniques to protect the transection plane, such as ligation of the main pancreatic duct, application of meshes, patches, falciform ligament flap, or fibrin glue, but with inconsistencies [15–18]. In this light, the International Study Group of Pancreatic Surgery (ISGPS) has recently claimed with a position statement that, currently, there is no specific surgical technique to eliminate the development of clinically relevant POPF [18].

Continuous irrigation of the peripancreatic area is a well-known and established treatment strategy for the management of infected necrotizing pancreatitis. Several studies demonstrated the effectiveness and the safety of this technique to treat necrotizing pancreatitis [19–22]. The rationale of continuous irrigation in acute pancreatitis consists of diluting exudative pancreatic fluids and local bacteria. As a result, the local lavage enables to break inflammation, remove necrotic material and associated bacterial load, and prevent fluid collection and subsequent erosion damage. These same issues are also crucial after elective pancreatic surgery. Here, the use of continuous irrigation as prophylactic strategy or as treatment in case of POPF and fluid collections has been not extensively investigated yet, and only a few papers have explored this alternative technique. However, considering the important impact of POPF on surgical outcomes, investigating new prevention and mitigation strategies is certainly worthwhile. Therefore, we aimed to systematically review the literature to examine the use of local continuous irrigation after elective pancreatic surgery to evaluate its usefulness and safety in the prevention and treatment of POPF.

## Materials and methods

**Fig. 1:**
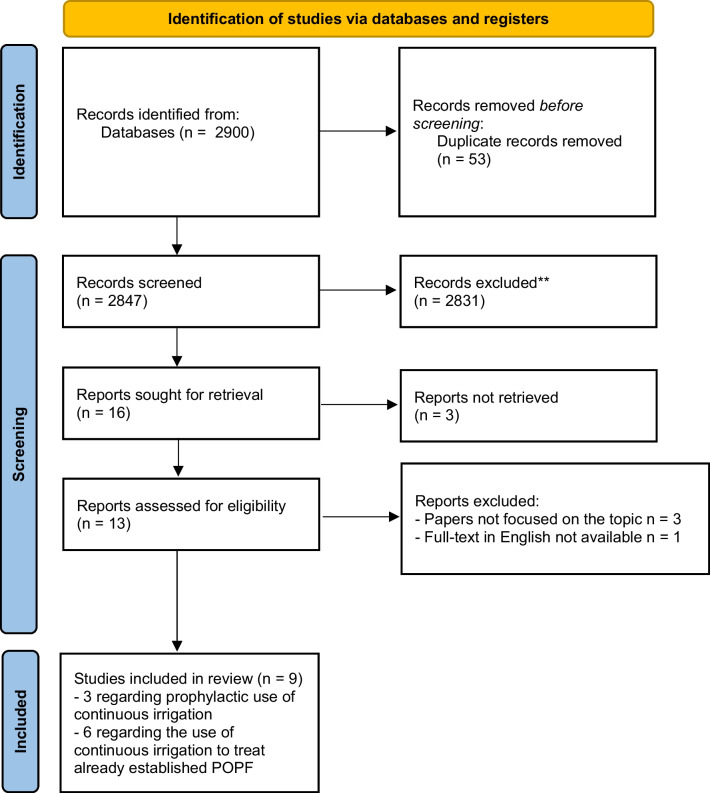
Flowchart according to the PRISMA guidelines 2020 showing the study selection

A systematic search of the literature was carried out on November 18th, 2022, screening the databases of Pubmed, Scopus, Web of Science, and Ovid MEDLINE. The following search strategy was used: (lavage OR irrigation) AND (((((((pancreatectomy) OR (pancreatoduodenectomy)) OR (pancreaticoduodenectomy)) OR (pancreatic surgery)) OR (pancreatic resection)) OR (pancreatic)) OR (pancreatic fistula)). The search strategy is shown in Fig. 1. We performed this systematic review according to the Preferred Reporting Items for Systematic Review and Meta-Analysis (PRISMA) guidelines (PRISMA checklist – Supplementary material 1) [23]. The study was pre-registered online on December 4th, 2022 (OSF Registry).

After duplicates’ exclusion, a review of titles and abstracts was conducted by 2 authors (I.P. and I.E.D.). Any disagreement between the two reviewers during the search and selection process was resolved by consensus. For all abstracts deemed potentially relevant, full texts were retrieved and reviewed. Studies providing data on continuous irrigation after pancreatic resection to prevent or treat pancreatic fistula, published in English language by peer-reviewed journals, were considered eligible and included in the systematic review. Abstract or congress articles, as well as systematic reviews, were excluded. No limitations were applied for histological characteristics, operation procedures, study designs, and methods used for the local lavage. As our systematic review focused on the use of continuous irrigation after elective pancreatic resections, articles dealing with the use of continuous lavage to treat acute pancreatitis were not considered. Papers reporting the use of drainage to treat POPF and fluid collections after elective pancreatic surgery, but without details regarding continuous lavage, were also excluded from the study. The number of excluded studies and the reasons for exclusion are described in the flowchart (Fig. 1). The following data were collected: author details, country, year of publication, recruitment period, study design, sample size, inclusion criteria, surgical technique, method used to perform continuous irrigation, and postoperative outcomes. The primary outcome was to evaluate the feasibility, efficiency, and safety of the procedure to prevent or treat POPF.

The Newcastle-Ottawa Scale quality assessment tool was used to assess the quality of the included cohort studies and potential bias [24]. Studies were rated from 0 to 9 stars, and those that received a score of 6 or above were considered as good/high quality. For the quality assessment of the included case reports, we used the Pierson evaluation scheme [25], resulting in a score with a maximum of 10 (a score ≥ 5 indicate a valid case report). The heterogeneity of the included articles did not enable any kind of statistical inference, and we decided to review the literature only descriptively.

## Results

### Study inclusion and quality assessment

From the initially identified 2900 studies, 9 studies provided data regarding continuous irrigation after pancreatectomy and met the inclusion criteria for the systematic review [26–34]. Table 1 shows the descriptive characteristics of the included studies. Six studies were retrospective, while 3 were case reports. In 3 studies, continuous irrigation was applied prophylactically to prevent the onset of POPF and related complications [26–28], whereas in the other 6 studies was used to manage a clinically relevant POPF and subsequent, already established fluid collections [29–34]. Of note, 3 articles reporting data regarding the prophylactic use of continuous local lavage after pancreatic surgery could not be retrieved and were excluded from the analysis [35–37]; the journal Hepatogastroenterology, in which all 3 articles were published between 2008 and 2010 was discontinued in 2015, and the papers are no longer available. The quality assessment of the included studies is reported in the Supplementary Table 2 (a-b).

**Table 1:** Basic characteristics of the studies included in the systematic review

Study	Country	Year of publication	Study design	Study period	N patients	Methods	Drain application/irrigation technique	Main result
Bu et al.	China	2013	Retrospective study	01/2005–01/2011	125 (60 irrigation vs 65 non-irrigation)	Prophylactic continuous irrigation around remnant pancreas after distal pancreatectomy	8F silicon tube for irrigation and two 24F to drain. Lavage immediately after surgery, 30–40 ml/h and adjusted overtime (1–2 l/day) for 7 days.	With irrigation: (1) lower clinically relevant POPF but not significant; (2) significantly lower POPF-related complications (3) no bleeding or reoperation
Adamenko et al.	Switzerland	2020	Retrospective study	09/2015–07/2019	21 (only irrigation, no comparison group)	Prophylactic continuous irrigation around remnant pancreas after distal pancreatectomy	A 15CH Salem sump irrigation tube near pancreatic stump and cystofix drainage in lower pelvis to drain. Irrigation started immediately and for 2POD** with 100 ml/h of Ringer solution, then reduce overtime until removal on POD7.	With irrigation: (1) only one case (8.3%) of POPF Grad B, (2) 2 (16.6%) patients had grade 3 or higher surgical complication, (3) no cases of reoperation or in-hospital mortality.
Chao et al.	China	2021	Retrospective study	02/2014–03/2019	50 (21 irrigation vs 29 non-irrigation)	Prophylactic continuous irrigation after limited pancreatic resection	Double catheterization cannula (10F drainage inside a 22F drainage) with negative pressure to drain and a 8F irrigation tube near pancreatic anastomoses or defects. Irrigation of normal saline at 1 ml/min. Drain removal on POD 5 or 7 after CT-scan without fluid collection and normal amylase.	With irrigation: (1) lower rate of clinically relevant POPF and POPF-related complications [irrigation: 11(37.9%) vs non-irrigation: 2 (9.5%), *P* < 0.05]; (2) length of stay significantly shorter after lavage (irrigation: 16.1 ± 6.3 days vs. non-irrigation: 20.6 ± 7.9 days, *p* = 0.039).
Hori et al.	Japan	2019	Case-report	Not reported	3 (only irrigation)	Continuous local lavage to treat intractable POPF*	Case 1: Two red vulcanized rubber tubes Case 2: one two-way Salem sump tube 14F Case 3: irrigation through transgastric tube. Irrigation with 1000 ml of saline per day until resolution.	With irrigation: (1) reduction of the duration of POPF, (2) no additional POPF related complications, (3) no reoperation.
Jiang et al.	China	2014	Case-report	Not reported	2 (only irrigation)	Enclosed passive infraversion lavage-drainage system to treat POPF grade C	Two Penrose drains (24CH) placed behind the anastomoses and one Penrose drain (24CH) in front of the pancreticojejunoanastomosis placed to different heights. Irrigation started on POD 7 and 5 respectively at 3–5 ml/min and stopped with amylase and bilirubin in the drain fluid tending to normal.	POPF resolution in both patients.
Wiltberger et al.	Germany	2015	Retrospective study	2005–2011	13 (only irrigation)	Continuous irrigation to treat POPF grade C and dehiscent pancreaticojejunostomy after pancreaticoduodenectomy	A 24F catheter for irrigation and two 20F Robinson drains to drain around the remnant pancreas. Irrigation with Ringer solution at 50-100 ml/h until to normalized infection parameter and stable clinical condition after lavage reduction.	With irrigation: (1) lower mortality (2 patients, 15%) compared to total pancreatectomy or preservation of the remnant pancreas without irrigation, (2) resolution of POPF in all other 11 patients, (3) preservation of endocrine function in 92%.
Lin et al.	China	2018	Retrospective study	2012–2018	34 (12 necrosectomy-drainage-irrigation vs. 22 only drainage without irrigation	Percutaneous endoscopic necrosectomy combined with percutaneous drainage and continuous irrigation to treat clinically relevant POPF	Implantation of a pigtail 18F under CT or ultrasound for the irrigation and percutaneous endoscopic necrosectomy by a choledochoscope every 3–4 days until recovery. Irrigation with normal saline at 1500 ml/day.	With necrosectomy and irrigation > lower incidence of postoperative delayed severe intraabdominal hemorrhage (31.8% vs. 0%; *p*=0.04).
Nakata et al.	Japan	2021	Retrospective study	01/2010–04/2020	47 (only irrigation)	Open drainage combined with negative pressure therapy and continuous irrigation to treat POPF grade B/C with fluid collection	Placement of 1 or 2 Penrose drains, then after removal negative pressure therapy and continuous irrigation. Irrigation method not specified.	POPF resolution in 98% of cases.
Bu et al.	China	2010	Case-report	01/2000–12/2008	3 (only irrigation)	Silicon tube in the pancreatic duct with continuous irrigation to treat dehiscent pancreaticojejunostomy after pancreaticoduodenectomy	Placement of a silicon tube in the pancreatic duct and continuous irrigation of the pancreatic stump with physiological solution at 20–40 ml/h.	With irrigation: (1) POPF resolution in all 3 cases, (2) no additional complication, (3) normal exocrine and endocrine function.

*POPF Postoperative pancreatic fistula; **POD postoperative day

### Prophylactic use of continuous irrigation after pancreatic surgery

Bu et al. first investigated the prophylactic use of continuous irrigation of the peri-pancreatic space after elective distal pancreatectomy (DP), comparing the surgical outcomes of 60 patients who underwent irrigation vs. 65 patients without irrigation [27]. The colleagues placed two 24F drains for the saline output, one close to the pancreatic stump and one in the left subphrenic fossa, and an 8F silicon tube tied to the drainage placed at the pancreatic stump for the irrigation. The local lavage was started immediately after the end of surgery and continued for 7 days, adjusting the liquid rate over time. The amylase content in the drain fluid was tested every 2 days, and the irrigation was stopped on the 7th postoperative day (POD) if the amylase value was normal and no signs of POPF were recognized. In all other cases, the lavage was continued. With the application of two outflow-drainages, no diffusion of fluids to other abdominal areas was experienced. Overall, POPF occurred in 31% of the patients, and this was clinically relevant (grade B or C) in 12%. The rate of clinically relevant POPF was 2-fold higher in the non-irrigation group, but not significant [irrigation: 5 (8%) vs. non-irrigation: 10 (15%), *p*=0.226]. In contrast, the rate of POPF-related complications and the overall incidence of intraabdominal complications was significantly lower after continuous irrigation of the pancreatic stump [irrigation: 23 (38%) vs. non-irrigation: 39 (60%), *p*=0.025]. Among the 60 patients who received continuous irrigation, abdominal fluid collection was observed only in one patient, and no cases of bleeding or reoperation were described. Moreover, these patients showed better clinical conditions and needed fewer specific treatments, such as interventional drainage, antibiotic treatment, or total parenteral nutrition, than those in the non-irrigation group. Given the promising results, the authors strongly recommended this technique in patients with high-risk factors for POPF, such as patients with soft pancreas or without ligation of the pancreatic duct during the operation.

The preventive continuous irrigation with passive drainage after distal pancreatectomy in patients at high risk of POPF was also recently promoted by Adamenko et al. [26]. The authors defined at high risk of POPF patients with thick pancreas at the site of transection (pancreas thickness at the approximate site of transection preoperatively measured on CT scan > 12 mm), soft pancreas texture, high body mass index (≥ 25 Kg/m^2^), and those who needed a multiorgan resection. Among 21 patients who underwent distal pancreatectomy, 12 patients were at high risk of developing POPF and received a 15-Ch Salem sump irrigation tube with passive drainage near the pancreatic stump (fixed to the peritoneum with 4-0 Vicryl Rapide) and a second drainage catheter in the lower pelvis. The irrigation was performed through the Salem drain starting immediately after the operation and continued for 2 POD with 100 ml/h of Ringer solution. The fluid was drained by passive gravity through the drainage in the pelvis. On POD 3, amylase and lipase were tested in the drain fluid, and when 3 times lower than the serum level, the irrigation speed was reduced to 50 ml/h. If amylase and lipase were still unremarkable on POD 4 and there were no clinical signs of POPF, the local lavage was interrupted, and only passive drainage was maintained until drains were both removed on POD 7. Among the 12 included patients, only one patient (8.3%) experienced a POPF of grade B, while overall 2 (16.6%) patients had severe surgical complications (grade 3 or more according to the Clavien–Dindo classification). No cases of reoperation or in-hospital death were reported. Accordingly, Adamenko et al. supported that irrigation can prevent the evolution of POPF from a simple biochemical leak to clinically relevant POPF and suggested its use in patients at high risk of POPF.

A third study group analyzed the use of prophylactic active irrigation drainage after limited pancreatic resection (central pancreatectomy, enucleation, and Beger procedure) and demonstrated a reduction of clinically relevant POFP and related complications [28]. Chao et al. compared the surgical outcomes of 29 patients who received close-suction drainage with 21 patients who underwent continuous irrigation. The irrigation was performed with saline solution flushed at a speed of 1 mL/min through an 8F tube, while for the outflow they placed a double catheterization cannula (composed of a 22 French outer catheter with a 10 French inner drainage tube) under negative pressure (2–4 kPa). The local lavage was begun after surgery, and the amylase value in the drain fluid was tested daily. The drains were removed on POD 5 or 7 after a CT scan excluding intraabdominal fluid collections and in case of unremarkable amylase level in the drain fluid. The authors showed that in the irrigation group, the rate of clinically relevant POPF and related complications was significantly lower (irrigation: 2 (9.5%) vs. non-irrigation: 11 (37.9%), *p*<0.05), and the mean postoperative length of stay was significantly shorter (irrigation: 16.1 ± 6.3 days vs. non-irrigation: 20.6 ± 7.9 days, *p*=0.039). As a result, Chao et al. suggested the prophylactic use of continuous active lavage as an effective alternative to improve the postoperative management after partial pancreatic resections.

### Use and results of continuous irrigation to treat clinically relevant POPF

Six studies reported the use of continuous irrigation to treat clinically relevant POPF and related complications after pancreatic surgery. Hori et al. presented three cases successfully treated by continuous local lavage [29]. In these three patients with intractable POPF, the intraoperatively placed drains were substituted with irrigation tubes, and continuous irrigation was performed with ca. 1000 ml of saline over 24 h. The drains were replaced by a fistulography performed through the drains themselves. After fewer days of treatment (4–7 days), a complete resolution of the POPF was observed, and the drainages were removed. Considering these promising results, the authors proposed an institutional protocol for the effective use of continuous local lavage to overcome POPF-related complications after pancreatic resections. With the same purpose, Jiang et al. proposed an alternative washing technique called “enclosed passive infraversion lavage-drainage system” (EPILDS), in which inlet (for the irrigation) and outlet tubes (for the drainage) are connected to bags placed at different heights at the bed of patients [30]. As the inlet tube was connected to a bag placed at the patient’s level while the outlet bag was positioned lower than it, the fluid flows outward passively due to a pressure gradient, and when the drain is obstructed, the fluid will backflow in the inlet tube avoiding intraabdominal fluid accumulation and dispersion. The authors applied this technique successfully in 2 patients with POPF. In another study, 13 patients with POPF grade C and dehiscent pancreaticojejunostomy underwent disconnection of the anastomosis and continuous irrigation of the preserved remnant pancreas [31]. In all patients, after the disconnection of the anastomosis the pancreatic duct was left open, except for one patient who received a pancreatic duct drainage. The jejunal limb was shortened and was closed near the choledochojejunostomy using a linear stapler. The local lavage was performed continuously with Ringer solution with a flow of 50–100 ml/h through a 24F catheter, and the fluid was drained by two 20F Robinson drainages, all placed near the pancreatic resection surface. Two patients (15%) died during the hospital stay. After a median irrigation of 25 days (range 21–61), all other POPFs had resolved and were no longer clinically relevant. Moreover, only one patient developed postoperative diabetes, while 10 patients had exocrine insufficiency. The authors concluded that in case of POPF grade C after pancreaticoduodenectomy, continuous irrigation and drainage of the remnant pancreas seems to be a simple and feasible alternative to total pancreatectomy, with lower mortality and preservation of the endocrine function. Similarly, Bu et al. presented 3 cases in which a dehiscent pancreaticojejunostomy was successfully treated with disconnection of the anastomosis, blind closure of the jejunal limb with a stapler or by suture, and the placement of a silicon tube in the pancreatic duct combined with continuous irrigation of the pancreatic stump [34]. In more detail, the distal part of the silicon tube was inserted into the lumen of the jejunal loop, to drain pancreatic secretions internally into the distal jejunum. Then, a fine catheter for postoperative irrigation and 2 drains for drainage were placed near the pancreatic stump and continuous irrigation with a physiologic solution was begun as soon as surgery was completed at 20–40 ml/h. The drains and irrigating catheter were removed in all 3 patients within 21–34 days after reoperation. Another alternative technique to treat fluid collection related to clinically relevant POPF after pancreatoduodenectomy was presented by Lin et al. [32]. They performed in 12 patients a percutaneous endoscopic necrosectomy (made by choledochoscope through a 2–3cm skin incision adjacent to the irrigation tube) combined with percutaneous drainage and irrigation and compared the outcomes with 22 patients who received percutaneous drain only. Patients treated with continuous irrigation had a lower incidence of postoperative delayed severe intraabdominal hemorrhage (31.8% vs. 0%; *p*=0.04), with no differences in terms of other complications. Recently, Nakata et al. also described a new procedure to treat POPF complicated with fluid collection based on open drainage combined with continuous negative pressure and irrigation [33]. In their work, among 605 patients who underwent pancreaticoduodenectomy, 95 (16%) developed a POPF grade B/C and 47 (8%) required open drainage. Here, the surgical wound was bluntly opened and 1 or 2 Penrose drains were placed through the fascia into the fluid collection. When the fluid outflow decreased, the drains were removed, and a continuous negative pressure therapy combined with continuous irrigation was performed through the open wound until the POPF resolution. The authors described a successful rate of 98% and, therefore, suggested this technique as a safe strategy for the management of complicated POPF after pancreaticoduodenectomy.

## Discussion

POPF represents for pancreatic surgeons still an unsolved and concerning issue to deal with [12, 18]. Data and surgeon’s perception identified the pancreas itself (i.e., soft pancreas with a small main pancreatic duct) as the most important risk factor for the development of POPF [2, 12, 38]. A specific surgical technique capable of eliminating or drastically reducing the development of clinically relevant POPF has not been identified yet [17, 18]. For this reason, every surgeon or institution has developed an “individual protocol” and uses a variety of techniques depending on the clinical situation, in the choice of which experience and not only evidence plays an important role [12]. Recently, we conducted a survey among experts in the field regarding the use of drains, and interestingly, 4 colleagues (9.5%) reported a proactive use of intraoperative placed drains for continuous irrigation of the remnant pancreas: one participant performs the local lavage routinely and the other 3 respondents only in selected cases. That means when a POPF is clinically highly suspected or prophylactically in presence of risk factors for POPF such as soft pancreas, very small main pancreatic duct, or difficult anastomosis. Continuous irrigation of the peripancreatic area is a diffusely accepted treatment strategy for the management of infected necrotizing pancreatitis [19–21]. By contrast, its use in pancreatic surgery, especially as a prophylactic strategy, is not well-known. In this regard, we reviewed the current literature to evaluate the use of continuous irrigation in elective pancreatic surgery.

In the included studies, the principle behind the use of continuous irrigation of the surgical area was to dilute the concentration of pancreatic secretion and the bacterial load, and quench the subsequent inflammatory process that leads to other complications like abscess, delayed gastric emptying, wound infection, sepsis, and erosion bleeding. This seems reasonable as Reuver et al. and Nahm et al. observed increased amylase concentration in the fluid derived intraoperatively around the surgical area during PD and DP, respectively. In both studies, the peripancreatic space was irrigated with 200-ml saline and then suctioned; after a while, 3 ml of fluid that reaccumulated around the pancreatic anastomosis or the pancreatic stump, respectively, was collected and analyzed. Both study groups demonstrated that the intraoperative amylase concentration in the fluid collected around the surgical area was highly predictive of POPF after surgery [39, 40]. On the same line, several studies in the last years have argued that acute pancreatitis occurs in the perianastomotic or transection area after pancreatic resection, and this is a trigger event for other postoperative complications, such as POPF and bleeding [41–45]. The importance of this phenomenon was confirmed by the ISGPS, which recently developed a consensus definition and grading of this specific entity, called “Postpancreatectomy Acute Pancreatitis” (PPAP) [46]. Regarding the bacterial load, our group recently found that, in bacterial swabs of drain fluid obtained from 108 patients with clinically relevant POPF (DP 47; PD 61), bacteria from the normal intestinal flora, particularly Enterobacterales, were frequently detectable (74% after PD and 34% after DP), and this correlated with more severe postoperative complications. How intestinal bacteria can colonize POPF-fluid is unclear, especially considering the frequent detection of them after DP, which is typically a “sterile” operation without opening of the intestinal lumen and without anastomosis, and that the pancreatic duct at the time of surgery is usually sterile [47, 48].

In this setting, it is not surprising that in 3 studies, the lavage was started immediately after the surgery and then adapted postoperatively until removal depending on clinical condition and amylase values in the drain fluids [26–28]. Although using different methods, in all 3 papers the surgical outcomes of the irrigation group were very encouraging in comparison with the non-irrigation group and data present in the current literature. Interestingly, the prophylactic use of continuous irrigation did not influence the overall rate of POPF, but, most importantly, reduced the occurrence of clinically relevant POPF and related complications, with subsequent shorter length of stay and lower mortality. In other words, continuous irrigation initiated immediately after the surgery does not seem to remove the risk of developing a biochemical leak (POPF grade A), but, diluting the pancreatic enzymes (and potentially also bacteria) in the surgical area, appears to prevent its evolution into a clinically relevant POPF (grade B/C). Therefore, patients at high risk of POPF may have a great benefit and seem the best candidate for this procedure.

Another possible application of continuous irrigation is to treat already established clinically relevant POPF, similarly to its use in necrotizing pancreatitis. In our systematic review, several papers presented alternative procedures with continuous irrigation that showed positive results in terms of POPF resolution and reintervention/reoperation rate. Although some of these procedures seem not so easy to reproduce and implement, this shows an increasing interest in the use of continuous irrigation after pancreatic resection with the intent to overcome POPF-related complications.

In our review, not only positive aspects but also concerns about the use of this technique were reported. There are two major issues on the use of irrigation mentioned in the included studies: (1) the risk of retrograde contamination and (2) the possibility of fluid diffusion to other abdominal areas, which may result in secondary fluid collection and infection. Bu et al. did not experience any complications with irrigation after distal pancreatectomy; however, they highlighted the importance of close observation of the drains [27]. In case of obstructions of the drains, they suggested performing some maneuvers to unclog them, and, if ineffective, to discontinue the flushing. In addition, they placed an additional drain in the left subphrenic space to reach the fluid accumulated by gravity, and in case of extended left pancreatectomy, they applied one more drain in the Winslow foramen. Similarly, Chao et al. did not observe irrigation-related complications; however, they emphasized the importance of taking preventive measures, such as maintaining drainage unobstructed and applying a filter for inflow air, to avoid fluid collection or contamination [28]. The feasibility and safety of continuous irrigation, as well as the increasing interest on this procedure in pancreatic surgery, are also demonstrated by the fact that recently two randomized controlled trials regarding the use of continuous irrigation after elective pancreaticoduodenectomy were registered in the WHO International Clinical Trials Registry Platform (ICTRP) and are currently ongoing (https://trialsearch.who.int/Trial2.aspx?TrialID=DRKS00025073, https://trialsearch.who.int/Trial2.aspx?TrialID=ChiCTR2100053135).

These systematic review presents several limitations. As explained above, despite in the search strategy, we did not apply limitations for histological features, surgical procedures, study designs, and irrigation methods. As such, we could retrieve only a few papers on this topic. Moreover, the heterogeneity between them did not allow any kind of statistical inference, but only a descriptive analysis. Certainly, this affects negatively the robustness of the reported results; however, the remarkable lack of knowledge on this topic in the current literature represents itself an important result of this study.

## Conclusion

In conclusion, although in the treatment of acute necrotizing pancreatitis continuous irrigation is considered helpful and safe, in the current literature, only a small number of publications are focused on its use after pancreatic resection to prevent or better manage a POPF. In our systematic review, the included studies showed promising results, and several study groups suggested the use of this technique, especially in patients at high risk of POPF. Further investigations and randomized trials are certainly needed to confirm these results.

### Supplementary informations

ESM 1 Supplementary Material 1. Checklist of the PRISMA Guidelines 2020 (DOCX 32 kb)

ESM 2 (DOCX 14 kb)
