# Supplementary material for: Continuous irrigation after pancreatectomy: a systematic review
Source: Langenbecks Arch Surg. 2023 Sep 2;408(1):348. doi: 10.1007/s00423-023-03070-5 (PMC10474975; doi:10.1007/s00423-023-03070-5)
Supplement: Supplementary file 2 — (DOCX 14 kb) [file 423_2023_3070_MOESM2_ESM.docx]

**Supplementary Table 2a. Details of the Newcastle-Ottawa Scale score for the included cohort studies.**

| **NOS items** | **Bu et al.** | **Adamenko et al.** | **Chao et al.** | **Wiltberger et al.** | **Lin et al.** | **Nakata et al.** |
| --- | --- | --- | --- | --- | --- | --- |
| Representativeness of the exposed cohort | * | * | * | * | * | * |
| Selection of the non- exposed cohort | * |  | * |  | * |  |
| Ascertainment of exposure | * | * | * | * | * | * |
| Demonstration that outcome of interest was not present at the start of the study | * | * | * |  |  |  |
| Comparability of cohorts on the basis of the design or analysis | ** |  | * |  | ** |  |
| Assessment of outcome |  |  |  |  |  |  |
| Follow-up was long enough for outcomes to occur | * | * | * | * | * | * |
| Adequacy of follow-up of cohorts | * | * | * | * | * | * |
| **Total Score** | **8** | **5** | **7** | **4** | **7** | **4** |

**Supplementary Table 2b. Details of the Pierson evaluation scheme for the included case reports.**

| **Evaluated Parameters** | **Bu et al.** | **Hori et al.** | **Jiang et al.** |
| --- | --- | --- | --- |
| Documentation (0-2) | 1 | 2 | 1 |
| Uniqueness (0-2) | 2 | 2 | 2 |
| Educational value (0-2) | 2 | 2 | 2 |
| Objectivity (0-2) | 2 | 1 | 1 |
| Interpretation (0-2) | 2 | 1 | 1 |
| **Total Score** | **9** | **8** | **7** |
